# Fusion of CT radiomics and autoantibody biomarkers for enhanced prediction of lung cancer diagnosis: a comprehensive study

**DOI:** 10.3389/fonc.2025.1591156

**Published:** 2025-10-29

**Authors:** Xianwu Xia, Jinshuo Liu, Yiman Du, Xiaowen Ma, Guoming Zhou, Jianjun Yuan, Qianjin Hua, Lingling Wang, Haitao Jiang, Caidi He, Chibo Liu

**Affiliations:** ^1^ Department of Oncology Intervention, Municipal Hospital Affiliated of Taizhou University, Taizhou, Zhejiang, China; ^2^ The First Clinical College, Wenzhou Medical University, Wenzhou, Zhejiang, China; ^3^ Clinical Medicine College, Nanchang Medical College, Nanchang, Jiangxi, China; ^4^ Department of Radiology, Fudan University Shanghai Cancer Center, Xuhui, Shanghai, China; ^5^ Department of Oncology, Fudan University Shanghai Cancer Center, Xuhui, Shanghai, China; ^6^ Department of Clinical Laboratory, Zhejiang Cancer Hospital, Hangzhou, Zhejiang, China; ^7^ Wenzhou Medical University, Wenzhou, Zhejiang, China; ^8^ Department of Radiology, Zhejiang Cancer Hospital, Hangzhou, Zhejiang, China; ^9^ Department of Nursing, Municipal Hospital Affiliated of Taizhou University, Taizhou, Zhejiang, China; ^10^ Department of Clinical Laboratory, Municipal Hospital Affiliated of Taizhou University, Taizhou, Zhejiang, China

**Keywords:** lung cancer, CT radiomics, autoantibody biomarkers, early diagnosis, predictive modeling, multimodal integration

## Abstract

**Introduction:**

Early and accurate diagnosis of lung cancer is crucial for improving treatment outcomes and patient survival. This study investigates the combined use of computed tomography (CT) radiomics and autoantibody biomarkers as a novel approach to enhance lung cancer diagnosis.

**Methods:**

We analyzed 258 patients from two centers, dividing into training, internal validation, and external validation cohorts. CT scans were standardized, and 1106 radiomic features were extracted. The recursive feature elimination method was applied to iteratively eliminate the redundant features. Autoantibody levels were assessed using a multiplex immunoassay targeting seven specific biomarkers. After resampling the training dataset by using synthetic minority over-sampling technique, the support vector machine classifier was employed to train classification models. We developed separate predictive models for CT radiomics and autoantibody testing and then fused the two models to evaluate performance.

**Results:**

The fusion model demonstrated significantly improved diagnostic accuracy, with area under the receiver operating characteristic curve (AUC) values of 0.90 ± 0.02, 0.83 ± 0.08, and 0.78 ± 0.09 in three cohorts, outperforming both the CT radiomics-only (AUC: 0.87 ± 0.03, 0.76 ± 0.10, 0.74 ± 0.10) and autoantibody-only models (AUC: 0.67 ± 0.06, 0.55 ± 0.15, 0.57 ± 0.10). Decision curve analysis indicated a higher net benefit of the integrated model across various threshold probabilities.

**Conclusion:**

The fusion of CT radiomics and autoantibody biomarkers significantly enhances the diagnostic performance for lung cancer. This integrated approach enhances early detection and reduces unnecessary interventions, paving the way for personalized treatment strategies. Future research should focus on clinical validation and optimization of this model to facilitate its implementation in routine clinical practice.

## Introduction

Lung cancer remains the leading cause of cancer-related mortality worldwide, accounting for nearly 1.8 million deaths annually (1). One of the primary reasons for its high mortality rate is the frequent diagnosis at advanced stages when curative treatment options are limited. Despite advancements in treatment, the prognosis for lung cancer patients remains poor, primarily due to the late-stage diagnosis of the disease. Early detection of lung cancer is crucial for improving survival rates, as it allows for timely intervention when the disease is still localized and more amenable to treatment. However, current diagnostic methods, including imaging techniques like low-dose computed tomography (CT) and sputum cytology, have limitations in sensitivity and specificity, often leading to false positives or missed early-stage tumors (2). Consequently, there is an urgent need for more accurate and reliable diagnostic tools that can detect lung cancer at an earlier stage and differentiate malignant from benign lesions (3).

Recent advancements in medical imaging and molecular diagnostics have opened new avenues for improving the accuracy of lung cancer detection. Among these, CT radiomics—the extraction and analysis of high-dimensional data from CT images—has gained significant attention. Radiomics enables the quantitative assessment of tumor characteristics, such as shape, texture, and intensity, which can provide insights into tumor heterogeneity and biology that are not discernible through traditional visual inspection (4–6). Studies have shown that radiomic features can differentiate between benign and malignant lung nodules, predict tumor aggressiveness, and even forecast treatment response and patient outcomes (7).

Complementing the radiomic approach, autoantibody testing represents a promising blood-based diagnostic method (8–10). Autoantibodies are produced by the immune system in response to tumor-associated antigens (TAAs), which are proteins expressed by cancer cells. The presence of specific autoantibodies in the blood can serve as early indicators of malignancy, potentially before the tumor is detectable by imaging (11–14). In lung cancer, several specific autoantibody biomarkers have been identified that are associated with the presence of the disease. These biomarkers provide a blood-based, minimally invasive diagnostic tool that can complement imaging findings and offer additional information about the underlying tumor biology.

While CT radiomics and autoantibody testing have each demonstrated potential in lung cancer diagnosis, their combined application has not been extensively explored. The fusion of radiomic features and autoantibody biomarkers offers a multi-dimensional approach, leveraging both the anatomical and functional information provided by imaging and the molecular insights from blood tests (15–18). This integrative strategy has the potential to improve early detection, reduce false positives, and provide a more comprehensive understanding of the disease, ultimately leading to better patient outcomes (19, 20).

This study aims to explore the potential of integrating CT radiomics with seven specific autoantibody biomarkers to develop a robust predictive model for lung cancer diagnosis. We hypothesize that this multimodal approach will outperform traditional diagnostic methods and individual modalities, offering a significant advancement in the early detection of lung cancer. In this study, we discuss the principles of CT radiomics and autoantibody testing, outline the methodology for their integration, and explore the clinical implications and challenges of this approach. By examining the synergistic effects of combining these modalities, we aim to contribute to the development of a more effective diagnostic strategy for lung cancer, ultimately improving patient outcomes.

## Materials and methods

### Patient cohort

We retrospectively collected 206 patients from Zhejiang cancer (ZJC) hospital and 52 patients from Taizhou municipal (TZM) hospital. The patients in ZJC hospital were randomly divided into a training cohort (N = 166) and a validation cohort 1 (N = 40) with a ratio of 80%:20%. The patients involved in TZM hospital were used as a validation cohort 2. Inclusion criteria are as follows: 1) patients with a confirmed diagnosis of lung cancer based on surgery histopathological examination. 2) patients with benign pulmonary conditions confirmed through surgery or biopsy histopathological examination. 3) availability of pre-treatment CT scans and serum samples. Exclusion criteria include: 1) incomplete imaging or biomarker data. 2) recent surgical or therapeutic interventions that may alter biomarker levels. This study was approved by the institutional review board of Taizhou Municipal Hospital (LWSL202400230) and Zhejiang Cancer Hospital (IRB-2022-625), and it complies with the Declaration of Helsinki. Informed consent was obtained from all participants.

**Figure 1:**
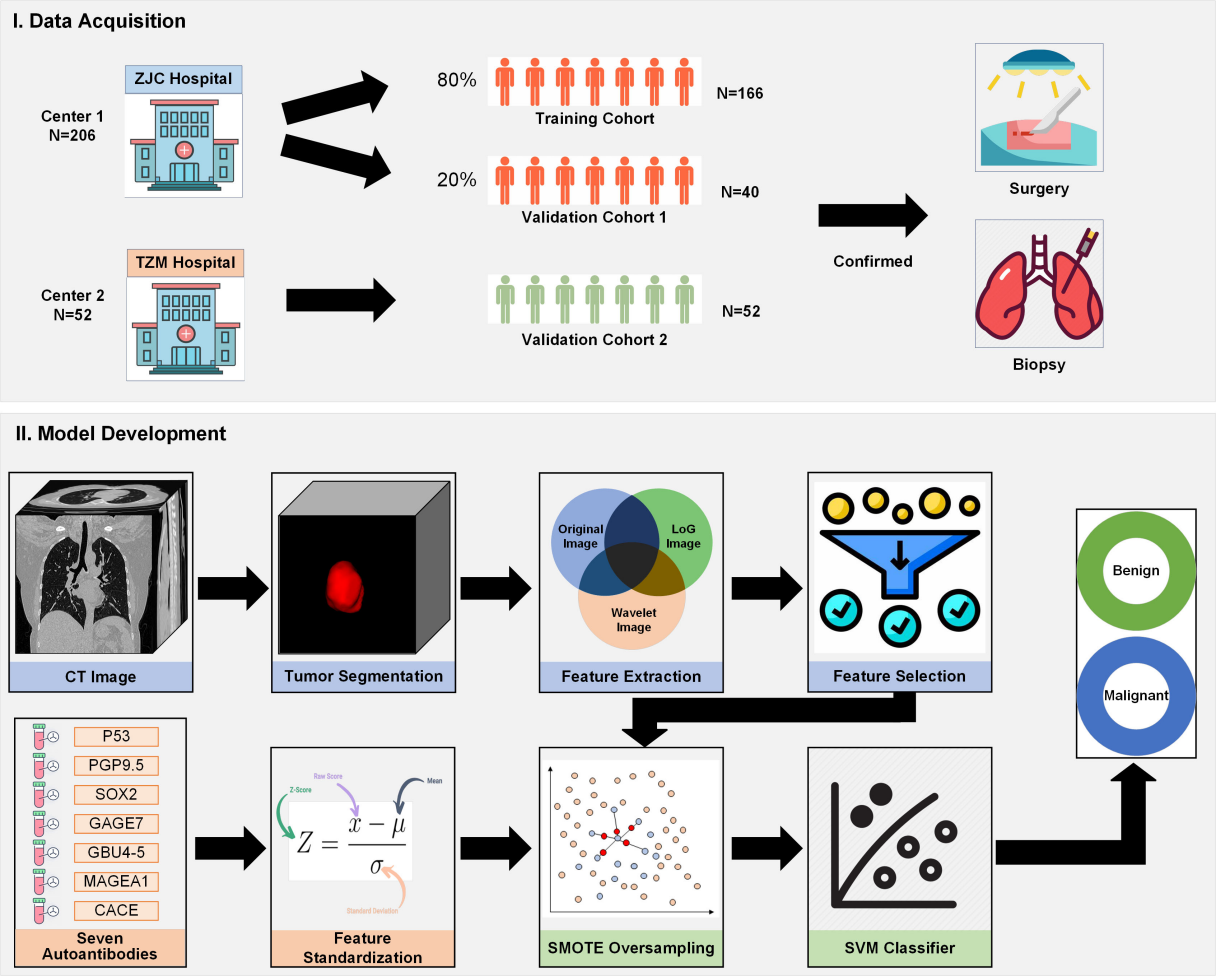
Flowchart of the study design, demonstrating patient selection, CT radiomics feature extraction, autoantibody biomarker testing, model development, and performance evaluation.

This study employs a retrospective cohort design to evaluate the effectiveness of integrating CT radiomics with autoantibody biomarkers for lung cancer diagnosis. The study involves the analysis of CT imaging data and corresponding serum samples from patients diagnosed with lung cancer and those with benign pulmonary conditions. The primary objective is to assess the diagnostic performance of the combined approach compared to traditional methods. **Figure 1** shown the flowchart of this study.

### CT imaging and radiomic feature extraction

#### Imaging protocol

CT scans were performed using scanners with manufacturers from Siemens, GE, Philips or United Imaging Healthcare at two centers. All patients underwent contrast, high-resolution chest CT imaging before any treatment or intervention. The imaging parameters used were standardized across all patients to ensure consistency in data acquisition and radiomic feature extraction. The tube voltage was 120 kVp and tube current was in 200–300 mAs using automated exposure control. The slice thickness was 1.0-5.0 mm. The field of view was adjusted to cover the entire thorax, from the lung apices to the costophrenic angles. Each CT slice was reconstructed with a matrix of 512×512 pixels.

#### Radiomic feature extraction

Tumor segmentation was performed manually by experienced radiologists (X. M.) with 6 years of experience. The process involved outlining the tumor boundaries on each CT slice where the lesion was visible, ensuring accurate delineation of the entire tumor volume. Manual segmentation was chosen to ensure high precision in capturing tumor heterogeneity and shape, critical for radiomic analysis. This segmentation procedure was carried out using ITK-SNAP software (http://www.itksnap.org/). The radiologist manually outlined the tumor on each axial slice of the CT scan. Care was taken to exclude surrounding structures, such as blood vessels, bronchi, and pleura, to focus on the tumor itself. After segmenting in the axial plane, the segmentation was cross-checked in coronal and sagittal planes to ensure consistency and accuracy of the tumor boundary across multiple views. To ensure reliability, a second radiologist independently reviewed a random subset of 20 segmented tumors. Discrepancies between radiologists were resolved through consensus discussion. The interobserver variability was measured using the Dice similarity coefficient, with a value of 0.805 indicating high agreement between the observers. All segmented tumors were rechecked by a senior radiologist (X. W.) with an experience of 12 years. This manual segmentation process provided high-quality, detailed delineations of the tumor, which are crucial for the subsequent extraction of radiomic features.

Following manual segmentation of the tumors, radiomic features were extracted from the segmented tumor regions using Pyradiomics (https://pypi.org/project/pyradiomics/) Python programming (21). Before feature extraction step, all the CT images were resampled with a new image spacing of (1mm, 1mm, 1mm). Radiomics transforms CT images into high-dimensional data by quantifying tumor characteristics such as shape, texture, and intensity, which reflect underlying pathophysiological traits. The feature extraction process adhered to the Imaging Biomarker Standardization Initiative guidelines to ensure reproducibility and comparability. A total of 1106 radiomics features were extracted to decode the imaging phenotypes of lung tumor initially. These extracted features were categorized into shape features, first-order intensity (histogram-based) features, texture features (second-order features) and higher-order features. Shape features quantify the three-dimensional geometry of the tumor, which may provide insights into its malignancy and invasiveness. First-order intensity features describe the distribution of voxel intensities within the tumor, providing an overview of the tumor’s internal composition. Texture features capture intra-tumoral heterogeneity by assessing the spatial distribution of intensity patterns. Higher-order features are derived from filtered images, which apply transformations (e.g., wavelet, Laplacian of Gaussian) to enhance specific image patterns. These transformations capture complex textural variations that may be linked to tumor biology.

### Radiomics model development

Before developing the radiomics model, all 1106 radiomic features were standardized using z-score normalization to ensure that all features had a mean of 0 and a standard deviation of 1. This normalization step prevented features with larger ranges from dominating the model. Due to the high dimensionality of radiomic features, dimensionality reduction was performed using recursive feature elimination to iteratively eliminate the least significant features, based on the performance of a support vector machine (SVM) classifier, until the optimal subset was identified (22). To prevent overfitting, especially in the presence of high-dimensional radiomic features, we used 10-fold cross-validation in conjunction with feature selection. This approach ensures that the selected features generalize well across different subsets of the data, enhancing the robustness of our model.

Given the imbalance in the dataset, with a relatively small number of benign cases compared to malignant cases, Synthetic Minority Over-sampling Technique (SMOTE) was applied to address this issue (23). Class imbalance can lead to biased predictions, where the model becomes overly inclined towards the majority class (malignant cases), reducing its ability to accurately detect benign cases. SMOTE generates synthetic samples of the minority class (benign tumors) to create a more balanced dataset, thus improving model performance. Using the imbalanced-learn library in Python, SMOTE was applied to the training data to synthetically increase the number of benign cases. The SMOTE function was called with default parameters (k=5) to oversample the benign class until it reached the same size as the malignant class, resulting in a balanced training set.

Finally, SVM with a linear kernel was utilized as one of the primary models to classify lung nodules as malignant or benign based on the extracted radiomic features. The choice of a linear kernel was motivated by the need for a simple, interpretable model that could effectively separate the two classes in high-dimensional feature space while avoiding overfitting.

### Autoantibody model development

Autoantibody data were collected from blood samples of patients included in this study. The concentration levels of each of the seven autoantibodies were measured and recorded. The selected seven autoantibodies which involve p53, PGP9.5, SOX2, GAGE 7, GBU4-5, MAGE A1, and CAGE, are well-established lung cancer biomarkers. p53 is a well-known tumor suppressor protein that plays a crucial role in regulating the cell cycle and initiating apoptosis. Mutations in the p53 gene are frequent in lung cancer, and the presence of anti-p53 antibodies has been associated with advanced stages of the disease and poor prognosis. PGP9.5 is a ubiquitin carboxyl-terminal hydrolase that is expressed in various tissues, including the nervous system and endocrine cells. In lung cancer, particularly small cell lung cancer, autoantibodies against PGP9.5 have been detected and may serve as a potential biomarker for early diagnosis. SOX2 is a transcription factor essential for maintaining pluripotency in stem cells and is involved in cellular differentiation. It is frequently overexpressed in lung cancer, and the presence of anti-SOX2 antibodies has been linked to tumor aggressiveness and patient survival. GAGE 7 is part of the GAGE family of cancer/testis antigens. The presence of autoantibodies against GAGE 7 in lung cancer patients has been reported and may be useful for serological diagnosis. GBU4–5 is another cancer/testis antigen that is aberrantly expressed in lung cancer. It is involved in cellular growth and survival, and the detection of anti-GBU4–5 antibodies can help in identifying lung cancer patients. MAGE A1 is a member of the melanoma-associated antigen family and is a cancer/testis antigen. It is not expressed in normal tissues except for the testes but is frequently found in lung cancer. CAGE is a cancer/testis antigen that is expressed in lung and esophageal cancers. It is involved in cellular growth regulation and the presence of anti-CAGE antibodies has been detected in lung cancer patients, indicating its potential as a serological marker. These biomarkers target TAAs and are often detectable in the early stages of lung cancer, making them valuable for diagnostic purposes.

These autoantibodies were chosen based on their relevance to lung cancer and previous evidence showing their diagnostic performance in differentiating malignant from benign lung conditions. Cases with missing autoantibody measurements were imputed using the median value of the corresponding biomarker within the dataset. Autoantibody levels were standardized using z-score normalization to ensure that all features contributed equally to the model training process. To avoid biases, the same SMOTE oversampling method and SVM classifier were used to develop autoantibody biomarker based model. The model is designed to leverage the diagnostic potential of seven specific autoantibodies known to be associated with lung cancer.

### Fusion of radiomics and autoantibody biomarkers

CT radiomics and autoantibody biomarkers provide distinct, non-overlapping information about lung cancer. The autoantibody biomarker panel provides complementary information to radiomic features, potentially enhancing the accuracy of lung cancer diagnosis. By fusing these two data types, the model benefits from both anatomical and molecular information, potentially increasing its ability to discriminate between benign and malignant lesions (24, 25). We employed a feature-level fusion strategy to integrate radiomics features and autoantibody biomarkers for lung cancer classification. Radiomics features extracted from CT images and the seven autoantibody biomarkers were concatenated into a single feature vector. Before fusion, both feature sets were normalized to ensure that radiomics features and autoantibody levels were on the same scale. Standardization was applied separately to each data modality, using z-score normalization. This feature-level fusion combines both types of information into a high-dimensional feature space, which is then used as input for the classification model. In this feature fusion model development process, the same SMOTE oversampling method and SVM classifier were also applied to avoid biases caused by different feature processing method.

### Model performance evaluation

The trained models, including the radiomics model, autoantibody model, and the fused radiomics-autoantibody model, were evaluated based on multiple performance metrics to thoroughly assess their predictive capabilities in diagnosing lung cancer. Both internal validation and external validation were performed to ensure the robustness of the models. To ensure the generalizability of the radiomics model, it was tested on an independent validation dataset consisting of new patients not included in the training process. The validation dataset helped confirm that the model could accurately predict lung cancer in diverse patient populations and imaging settings.

In addition to the standard performance metrics such as accuracy (ACC), sensitivity (SEN), specificity (SPE), positive predictive value (PPV), and negative predictive value (NPV), Odds Ratio (OR) was calculated to assess the strength of association between the predicted outcome and the actual diagnosis. The area under the receiver operating characteristic (ROC) curve (AUC) measures the model’s ability to distinguish between malignant and benign cases. A higher AUC score indicates better discriminatory power, with a score of 1.0 representing perfect classification. A comprehensive measure of model performance, capturing both sensitivity and specificity across different thresholds.

Comparisons between diagnostic performance metrics and traditional methods were conducted using appropriate statistical tests, such as paired t-tests or chi-square tests. To correct for multiple comparisons, we employed the Bonferroni method. Confidence intervals for all performance metrics were calculated using bootstrap resampling. The significance testing between models was performed using DeLong’s test for AUC comparisons, ensuring the statistical validity of our results. The statistical significance level was set at p < 0.05, with any p-values below this threshold indicating significant differences between models or compared to traditional diagnostic methods. We developed and implemented our models using Python programming software version 3.9.0 (https://www.python.org/). The Python packages used in our study include SimpleITK, scikit-image, numpy, pyradiomics, scikit-learn, and scipy. Default parameters in these packages were utilized, ensuring straightforward application and validation in future studies. All software tools used in our study are compatible with the data format used in the literature, ensuring the validity of our conclusions.

## Results

### Patient demographics and clinical characteristics

A total of 258 patients were included in the study, comprising 222 patients with confirmed lung cancer and 36 patients with benign pulmonary conditions. The clinical and demographic characteristics of the three cohorts are summarized in **Table 1**. The histological diagnosis and seven autoantibodies distribution in three cohorts was illustrated in **Supplementary Table 1**.

**Table 1:** Summary of patient demographics and clinical characteristics in the three cohorts.

Characteristic	Training cohort (N=166)	Validation cohort 1 (N=40)	Validation cohort 2 (N=52)
Sex			
Female	82 (49.40%)	21 (52.50%)	29 (55.77%)
Male	84 (50.60%)	19 (47.50%)	23 (44.23%)
Age	58.98±10.96	58.83±11.20	63.75±12.64
Pathology			
Benign	23	5	8
Malignant	143	35	44
Autoantibody			
p53	2.78±9.87	2.07±3.68	3.60±25.71
PGP9.5	0.52±1.52	0.24±0.49	4.87±25.69
SOX2	3.79±7.71	6.07±17.09	3.44±13.74
GAGE 7	6.61±21.35	2.77±3.30	3.93±13.81
GBU4-5	1.84±3.82	1.95±4.52	2.73±7.05
MAGE A1	2.19±8.02	1.58±5.14	2.78±8.15
CAGE	0.74±3.31	0.98±2.44	1.34±6.02

### CT radiomics analysis

**Figure 2:**
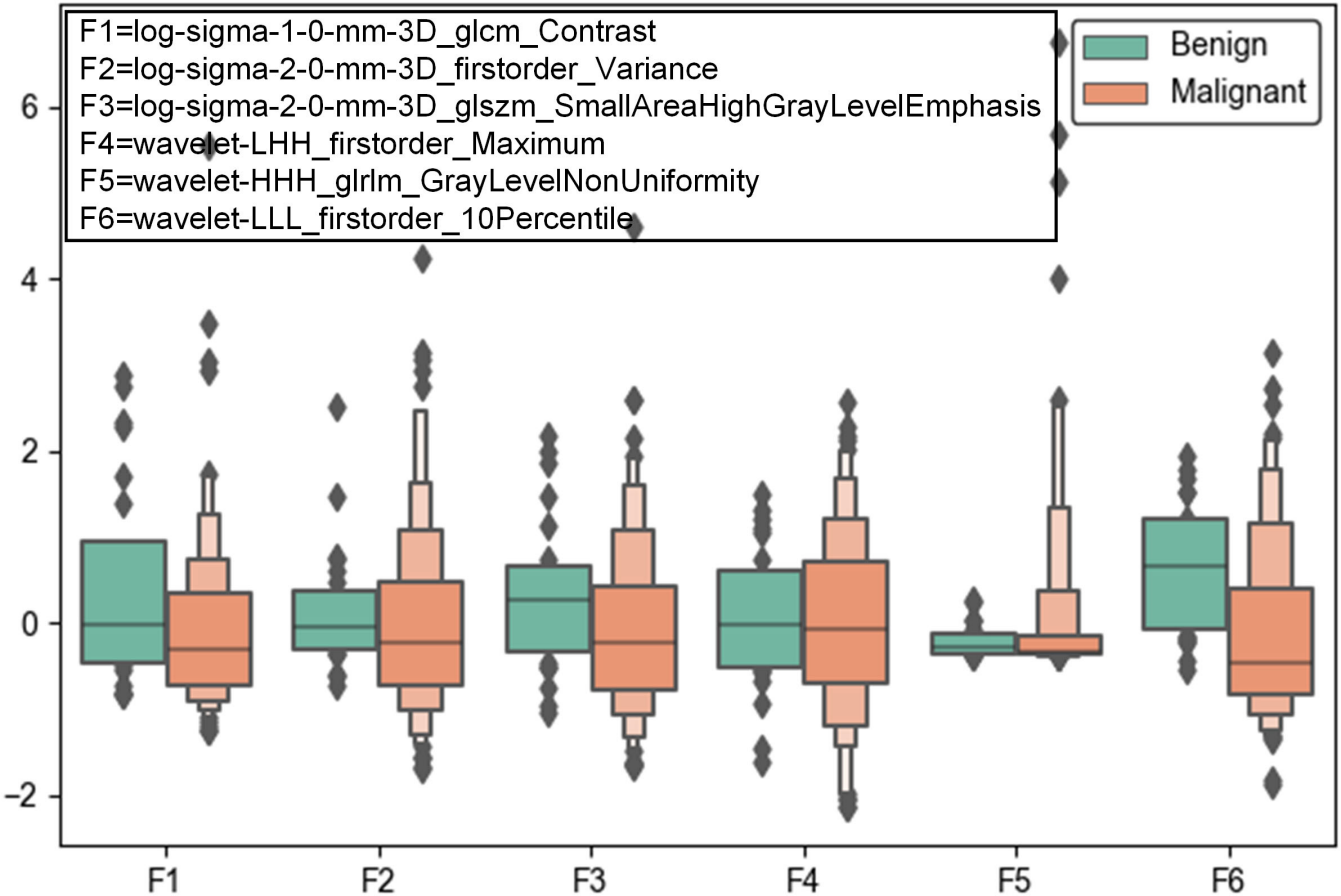
Box plots of the top six significant radiomic features between malignant and benign pulmonary lesions.

A total of 1106 radiomic features were extracted from CT scans, including texture, shape, intensity features, wavelet features and LoG features. Six features such as log-sigma-1-0-mm-3D_glcm_Contrast, log-sigma-2-0-mm-3D_firstorder_Variance, log-sigma-2-0-mm-3D_glszm_SmallAreaHighGrayLevelEmphasis, wavelet-LHH_firstorder_Maximum, wavelet-HHH_glrlm_GrayLevelNonUniformity, wavelet-LLL_firstorder_10Percentile demonstrated significant differences between malignant and benign lesions (see **Figure 2**). It can be seen that wavelet features and LoG features play a vital role in distinguish between benign and malignant tumors.

### Model performance

**Figure 3:**
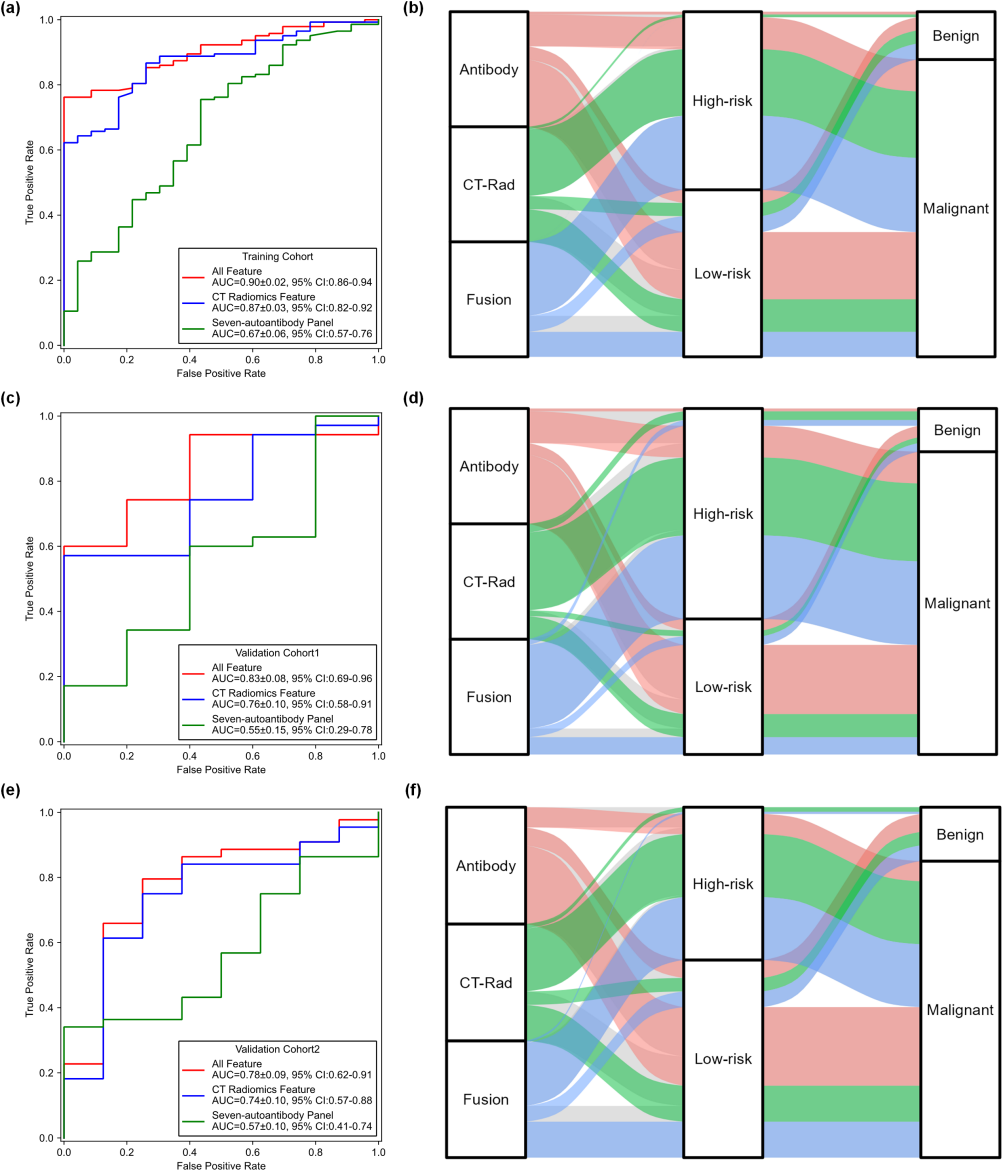
Comparisons of performance generated by three models across three cohorts. **(a, c, e)** ROC curves comparing the performance of the CT radiomics-only model, autoantibody-only model, and fusion radiomics-autoantibody model across three cohorts. **(b, d, f)** Sankey diagrams showing the predicted classification results of malignant and benign cases for the three models across the training cohort, validation cohort 1 and validation cohort 2, illustrating the distribution of cases between the prediction categories.

The ROC curves, AUC values and corresponding 95% confidence intervals (CIs) for the CT radiomics-only model, autoantibody-only model, and the fused radiomics-autoantibody model in training cohort and two validation cohorts are shown in **Figures 3a-c**. The fused model consistently outperformed the individual radiomics and autoantibody models across all cohorts, achieving the highest AUC values of 0.90 ± 0.02 (95% CI: 0.86-0.94), 0.83 ± 0.08 (95% CI: 0. 69-0.96), and 0.78 ± 0.09 (95% CI: 0.62-0.91) in three cohorts, respectively. The fusion model demonstrated significantly higher AUC values compared to the individual models, with P < 0.05, indicating that the combination of CT radiomics and autoantibody biomarkers greatly enhances diagnostic performance.

Meanwhile, the CT radiomics-only model also performed well, but it was less effective than the fused model. The CT radiomics model yielded a higher AUC values of 0.87 ± 0.03 (95% CI: 0.82-0.92), 0.76 ± 0.10 (95% CI: 0. 58-0.91), and 0.74 ± 0.10 (95% CI: 0.57-0.88) than that of autoantibody model in three different cohorts (P<0.05). While the radiomics model yielded reasonably high AUC values, it was consistently outperformed by the fused model, demonstrating the added value of integrating biomarker information. These results provide robust evidence that combining radiomics features with autoantibody biomarkers significantly improves lung cancer diagnostic accuracy and reliability.

**Figures 3d-f** illustrates Sankey diagrams representing the flow of predicted results from the three models (radiomics-only, autoantibody-only, and fused radiomics-autoantibody models) across the three cohorts (training, validation cohort 1, and validation cohort 2). These diagrams offer a visual representation of how patients were classified as malignant or benign by each model and the overlaps or differences in predictions between the models. The fused model consistently shows less flow towards misclassifications, particularly in distinguishing benign conditions from malignant tumors, which are critical for accurate lung cancer diagnosis. These visualizations provide clear evidence of the benefits of combining CT radiomics and autoantibody biomarkers, as the fused model outperforms the individual models in all three cohorts.

**Table 2** summarizes and compares the performance metrics, including ACC, SEN, SPE, PPV, NPV value and OR, for the three models evaluated using the training cohort and two validation cohorts. The results in **Table 2** reflect the same trends as observed in the ROC curves. The fused radiomics-autoantibody model consistently yielded higher ACC, SEN, SPE, PPV, and NPV values than either the radiomics-only or autoantibody-only models. The OR further supports the robustness of the fused approach, indicating a stronger association with lung cancer diagnosis. These findings underscore the efficacy of integrating CT radiomics and autoantibody biomarkers in enhancing the overall diagnostic performance for lung cancer, making a compelling case for the adoption of multimodal approaches in clinical settings for early detection and accurate diagnosis.

**Table 2:** Comparison of model performance metrics for the CT radiomics-only, seven autoantibody-only, and fusion models across the training cohort and two validation cohorts.

Model	Dataset	ACC	SEN	SPE	PPV	NPV
CT Radiomics	Training Cohort	69.28 [62.05, 76.51]	67.13 [59.29, 74.64]	82.61 [60.87, 95.00]	96.00 [90.22, 98.97]	28.79 [18.84, 40.62]
	Validation Cohort 1	72.50 [57.50, 85.00]	77.14 [60.00, 88.57]	40.00 [0.00, 100.00]	90.00 [73.79, 96.97]	20.00 [0.00, 60.00]
	Validation Cohort 2	65.38 [50.00, 75.00]	63.64 [47.62, 76.19]	75.00 [28.57, 100.00]	93.33 [72.41, 97.06]	27.27 [11.11, 48.26]
Seven-autoantibody Panel	Training Cohort	59.04 [51.20, 66.27]	58.74 [50.35, 66.43]	60.87 [39.13, 80.00]	90.32 [82.83, 95.40]	19.18 [10.97, 29.58]
	Validation Cohort 1	60.00 [45.00, 75.00]	60.00 [42.86, 75.00]	60.00 [0.00, 100.00]	91.30 [73.68, 100.00]	17.65 [4.76, 42.86]
	Validation Cohort 2	44.23 [28.85, 55.77]	36.36 [21.28, 50.00]	87.50 [33.33, 100.00]	94.12 [61.60, 100.00]	20.00 [8.82, 36.67]
All Feature	Training Cohort	78.92 [72.29, 84.94]	79.02 [71.63, 85.00]	78.26 [57.14, 93.10]	95.76 [90.73, 98.37]	37.50 [24.00, 52.00]
	Validation Cohort 1	82.50 [67.50, 92.50]	85.71 [70.97, 94.44]	60.00 [0.00, 100.00]	93.72 [79.23, 100.00]	37.50 [0.00, 80.00]
	Validation Cohort 2	67.31 [51.92, 76.92]	65.91 [50.00, 78.57]	75.00 [28.57, 100.00]	93.55 [74.26, 97.22]	28.57 [11.11, 50.00]

**Figure 4:**
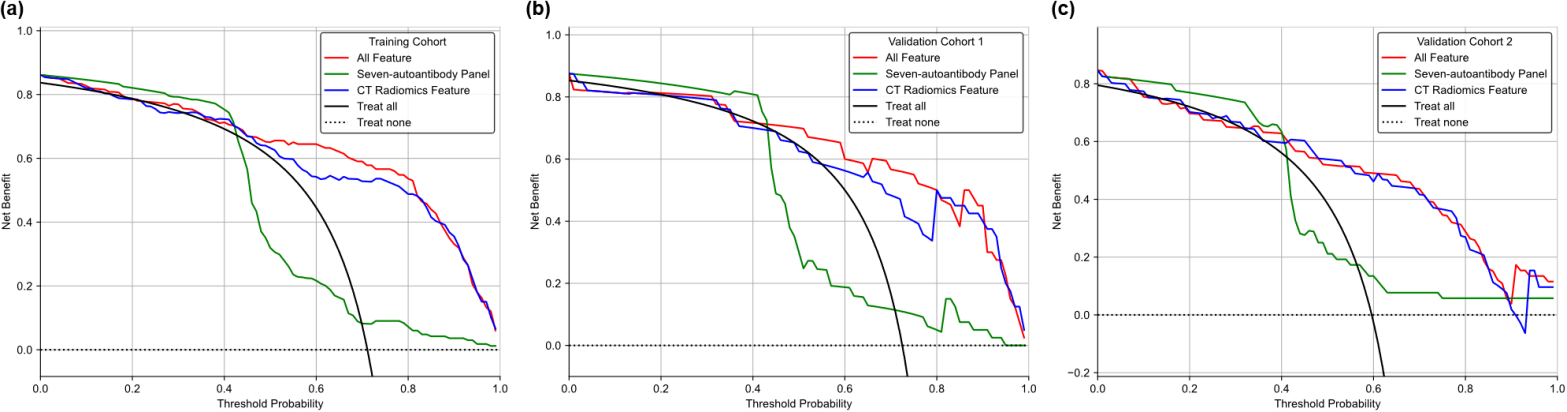
DCA comparing the clinical utility of the CT radiomics-only model, autoantibody-only model, and fusion model in three cohorts. **(a)** Training cohort; **(b)**Validation cohort 1; **(c)** Validation cohort 2.

To further validate the clinical value of the different models, a Decision Curve Analysis (DCA) was conducted. **Figure 4** presents the DCA curves for three models across the three cohorts. DCA provides a quantitative method to evaluate the clinical usefulness of predictive models by assessing the net benefits at varying threshold probabilities. The DCA curve for the fused model consistently lies above the other two models across all cohorts. This indicates a higher net benefit for clinicians at a range of threshold probabilities, emphasizing its superior clinical applicability in lung cancer diagnosis. The findings from the DCA highlight the practical implications of using the fused radiomics-autoantibody model in clinical settings. By demonstrating a higher net benefit across various threshold probabilities, this model supports more accurate and reliable decision-making in lung cancer diagnosis. The DCA results reinforce the importance of multimodal approaches, suggesting that integrating CT radiomics with autoantibody biomarkers can significantly enhance clinical outcomes.

## Discussion

This study investigated the integration of CT radiomics and autoantibody biomarkers for enhancing the prediction and diagnosis of lung cancer. Our results indicate that combining these two modalities significantly improves diagnostic accuracy compared to using either approach alone. The integrated model achieved superior performance metrics, including increased accuracy, sensitivity, and specificity, highlighting its potential for more reliable early detection of lung cancer. This integration represents a significant step forward in multimodal fusion models, as it captures both imaging features and molecular data, enabling a more comprehensive assessment of the disease. Previous models, while valuable, may not fully capture the complexity of lung cancer. By incorporating autoantibody biomarkers, our model leverages the immune system’s ability to recognize and respond to tumor-specific antigens, thereby enhancing diagnostic accuracy and clinical benefit. Compared to prior models, our approach offers a more robust and holistic assessment, particularly for early-stage lung cancer detection. Our study has several characteristics.

The fusion of CT radiomic features with autoantibody biomarkers provides a comprehensive approach to lung cancer diagnosis. The integrated model outperformed both the CT radiomics-only and autoantibody-only models, with significant improvements in metrics such as accuracy, sensitivity, and specificity. This suggests that the combination of imaging and serum biomarkers can capture a more holistic view of tumor characteristics and the body’s immune response, leading to improved diagnostic outcomes. The higher sensitivity of the fused model underscores its potential utility in early detection strategies. Early diagnosis is crucial for improving treatment outcomes and survival rates in lung cancer patients, and our results suggest that this integrated approach could facilitate timely interventions (26, 27). The results from DCA reinforce the clinical relevance of our findings. The integrated model exhibited a higher net benefit across a range of threshold probabilities, indicating its potential utility in guiding clinical decision-making. Current guidelines from the National Comprehensive Cancer Network (NCCN) and European Society for Medical Oncology (ESMO) recommend the use of CT scans and molecular tests for lung cancer diagnosis and treatment planning. However, these guidelines do not integrate the use of autoantibody biomarkers, which can provide additional information about the immune response to the tumor. Our approach complements the existing guidelines by potentially improving diagnostic accuracy and enabling early detection of lung cancer. Current guidelines (NCCN, ESMO) prioritize imaging (low-dose CT) and biopsy for lung cancer diagnosis. However, our fusion model aligns with emerging trends advocating non-invasive biomarkers for early detection. Unlike guidelines that focus on single modalities, our approach integrates radiomics and autoantibodies (e.g., GAGE7, CAGE) to improve specificity, addressing limitations of standalone low-dose CT.

The enhanced diagnostic accuracy achieved through the combined approach has significant clinical implications. Early and accurate diagnosis of lung cancer can lead to timely intervention and improved patient outcomes. The integrated model could potentially reduce false positives and negatives, thus minimizing unnecessary biopsies and follow-up procedures. Furthermore, this approach may aid in risk stratification, allowing for personalized treatment plans based on the individual characteristics of the patient’s disease. The fusion of CT radiomics with autoantibody biomarkers represents a promising advancement in lung cancer diagnostics. The combined model not only improved diagnostic performance but also demonstrated the complementary nature of these modalities. Radiomics provides detailed spatial and textural information from imaging data, while autoantibody testing offers insights into the body’s immune response to cancer. Integrating these two sources of data allows for a more comprehensive evaluation of the disease, capturing both structural and molecular features. Our fusion model of CT radiomics and autoantibody biomarkers could potentially reduce healthcare costs by improving the accuracy of lung cancer diagnosis. Early detection and accurate staging can lead to more effective treatment and better patient outcomes, ultimately reducing the overall burden on the healthcare system. The cost of performing CT scans and autoantibody tests is relatively low compared to the cost of advanced treatment options for late-stage lung cancer. Therefore, our approach may be economically favorable by enabling timely and accurate diagnosis.

CT radiomics offers a detailed analysis of tumor characteristics through the extraction of quantitative features from imaging data by providing a wealth of quantitative features derived from imaging data that can capture the subtle variations in tumor texture, shape, and intensity. Our study found that several radiomic features, such as wavelet features and LoG features, were significantly different between malignant and benign lesions. These features are reflective of underlying tumor heterogeneity and texture, which are contribute to the model’s ability to differentiate between lung cancer and benign conditions, supporting previous findings that radiomics can enhance tumor characterization and classification. The improved performance of the radiomics model underscores its utility in identifying subtle imaging characteristics that may not be evident through conventional visual assessment alone.

Autoantibody testing serves as a valuable complementary diagnostic approach to imaging-based diagnostics by detecting immune responses against TAAs. Our findings show that seven autoantibodies were present at higher levels in lung cancer patients compared to those with benign conditions. This aligns with existing literature that suggests autoantibody profiles can reflect the presence of cancer and potentially reveal disease-specific biomarkers (28). This supports the role of autoantibodies as biomarkers for lung cancer, offering a minimally invasive method for detecting tumor-associated immune responses. The incorporation of these biomarkers into diagnostic models provides additional molecular insight that can enhance the overall diagnostic process.

Despite the promising results, our study also has several challenges and limitations. First, variability in imaging protocols and biomarker assays across different institutions can affect the generalizability of the findings. Standardization of imaging and testing procedures is essential for consistent results. Although efforts were made to standardize imaging parameters and biomarker measurements across centers, unavoidable inter-scanner and inter-laboratory variability may still exist. In future studies, we aim to implement feature harmonization techniques such as ComBat for multi-center radiomics data and establish cross-laboratory standardization protocols for biomarker testing. Second, the study’s retrospective nature and sample size may limit the robustness of the findings. Although two validation cohorts were used, the total number of benign cases remains small, which could affect the generalizability of our findings. The relatively small number of benign cases was mainly due to the clinical prevalence of malignant pulmonary lesions in the enrolled centers. This imbalance is a limitation of the study, and its implications should be considered when interpreting the results. To mitigate this, we employed statistical resampling strategies (including SMOTE) to balance the training process and avoid model bias. While we used SMOTE to mitigate this issue, we recognize that oversampling cannot fully replicate the diversity of benign lesions. Larger, prospective studies are needed to validate these results and assess the model’s performance in diverse populations. Third, the integration of multiple data types requires sophisticated algorithms and may pose challenges in terms of computational complexity and model interpretability. Simplified models that balance performance with practicality are needed for clinical implementation. Future research should focus on addressing the limitations identified in this study. As the current study is retrospective, prospective validation is crucial for assessing clinical utility. Prospective studies with multi-center larger cohorts are necessary to validate the integrated model and refine its performance. Additionally, exploring other biomarker panels and imaging modalities could further enhance diagnostic capabilities (29). Advances in machine learning and artificial intelligence may also contribute to developing more efficient and interpretable models for clinical use.

## Conclusion

In conclusion, our study underscores the potential of combining CT radiomics and autoantibody biomarkers to improve lung cancer diagnosis. The fusion of CT radiomics and autoantibody testing represents a significant advancement in lung cancer diagnosis. This integrated approach can enhance early detection, minimize unnecessary procedures, and pave the way for personalized treatment strategies, ultimately contributing to better patient outcomes. Continued research and clinical validation will be crucial for optimizing this integrative strategy and translating its benefits into routine clinical practice.

## Data Availability

The original contributions presented in the study are included in the article/**Supplementary Material**. Further inquiries can be directed to the corresponding author/s.
