# Supplementary material for: Fusion of CT radiomics and autoantibody biomarkers for enhanced prediction of lung cancer diagnosis: a comprehensive study
Source: Front Oncol. 2025 Oct 29;15:1591156. doi: 10.3389/fonc.2025.1591156 (PMC12604974; doi:10.3389/fonc.2025.1591156)
Supplement: Supplementary file 1 [file Table1.docx]

**Supplemental Table 1:** Histological Diagnosis and Molecular Tests

| Autoantibody | Training Cohort | | | Validation Cohort 1 | | | Validation Cohort 2 | | |
| --- | --- | --- | --- | --- | --- | --- | --- | --- | --- |
|  | Benign | SCLC | NSCLC | Benign | SCLC | NSCLC | Benign | SCLC | NSCLC |
| p53 | 1.38±1.25 | 3.30±1.77 | 3.00±10.71 | 1.26±0.90 | - | 2.19±3.90 | 0.00±0.00 | 0.00±0.00 | 4.93±29.97 |
| PGP9.5 | 0.76±1.87 | 0.90±1.13 | 0.47±1.46 | 0.70±1.20 | - | 0.17±0.19 | 0.92±0.95 | 3.45±3.35 | 6.20±29.92 |
| SOX2 | 3.58±9.64 | 10.20±8.84 | 3.69±7.26 | 16.32±29.63 | - | 4.61±13.82 | 0.80±0.69 | 15.45±15.35 | 3.67±15.35 |
| GAGE 7 | 3.40±6.20 | 18.07±14.64 | 6.89±22.91 | 2.50±1.97 | - | 2.81±3.45 | 1.41±0.98 | 0.95±0.55 | 4.88±16.04 |
| GBU4-5 | 1.33±2.07 | 9.77±6.91 | 1.75±3.77 | 0.42±0.37 | - | 2.17±4.79 | 1.23±0.94 | 2.65±1.25 | 3.26±8.15 |
| MAGE A1 | 5.90±17.39 | 0.47±0.45 | 1.62±4.88 | 0.76±0.64 | - | 1.69±5.48 | 1.53±1.78 | 0.80±0.40 | 3.35±9.42 |
| CAGE | 0.24±0.44 | 0.13±0.05 | 0.83±3.59 | 0.16±0.12 | - | 1.69±5.48 | 0.27±0.17 | 0.20±0.10 | 1.75±7.00 |

Note: SCLC=small cell lung cancer, NSCLC=non-small cell lung cancer.
